# Characteristic visual phenotypes in Korean wild mice (KWM/Hym)

**DOI:** 10.1186/s42826-024-00230-6

**Published:** 2024-12-24

**Authors:** Munkhdelger Jamiyansharav, Haesol Shin, Boyoung Kim, Hongkyung Kim, Soo Jung Han, Je Kyung Seong, Jun Gyo Suh, Kyoung Yul Seo

**Affiliations:** 1 Department of Ophthalmology, Yonsei University College of Medicine, 50 Yonsei-ro, Seodaemun-gu, Seoul, 03722 Republic of Korea; 2 Korea Mouse Sensory Phenotyping Center (KMSPC), Yonsei University College of Medicine, Seoul, Korea; 3 Department of Medical Genetics, College of Medicine, Hallym University, 1, Hallymdaehak-gil, Chuncheon-si, 24252 Gangwon-do Republic of Korea; 4 Laboratory of Developmental Biology and Genomics, College of Veterinary Medicine, and Korea Mouse Phenotyping Center, Seoul National University, Seoul, Korea

**Keywords:** Korean wild mouse, KWM/Hym, Visual phenotyping

## Abstract

**Background:**

In the last few decades, numerous efforts have been made to develop a better mouse model to overcome the current limitations of laboratory inbred mouse models such as have a weaker and simpler immune status. As part of these efforts, in Korea, the Hallym university medical genetics research team has been developing a new inbred strain of Korean wild mouse KWM/Hym. It was suggested that this strain, which is derived from wild mice, might be useful for genetic research and may become a valuable tool for overcoming some limitations seen in inbred mice that are currently used in the laboratory. Furthermore, for this study, we aimed to determine the visual phenotype of this unique strain KWM/Hym, and consider whether and if they are suitable for visual research. To analyze their visual phenotype, we performed the functional and morphological examinations in KWM/Hym mice and compared the results with laboratory mice which are the most common background strain.

**Results:**

KWM/Hym had a thin corneal phenotype, thin but well-ordered retina due to their light body weight characteristic, and normal visual function similar to control mice. Unexpectedly, the KWM/Hym mice developed cataracts only at around 25 weeks old.

**Conclusions:**

We suggest Korean wild mouse KWM/Hym is useful for visual experiments and could be an animal model of eye disease in humans.

## Background

House mice (also known as *Mus musculus*) may have the widest geographical distribution of any mammalian species with the exception of humans. Their geological origin is likely in present-day India and the expansion to the periphery of the Eurasian range, and to the rest of the world, is related to human migration and activities. Then, the breeding of house mice has begun in the late 19th and the use of them as laboratory animals with the study of genetics by scientists was in the early 20th century [1, 2].

At present, they are the most widely studied and commonly used primary model organisms in biomedical research because they are economical and easy to handle, and in particular, over 90% of mouse genes share functions with the genes in humans. These similarities help scientists to gain information about the physiology and genetics of health and disease in the human body system, including the visual system. However, the laboratory-inbred mice have been genetically isolated from thcieir free-living relatives and too far removed from natural environmental conditions for many years. They live in standard facility room [3] controlled by temperature, humidity, light cycles and contaminants, even breathe filtered air, so there are some arguments that mice cannot be expected to mimic humans’ phenotypes because humans do not [4–6].

Meanwhile, over the last years, several review articles have highlighted the benefits of a wild mouse. They have discussed the demands for continued research on wild mice and efforts to create new inbred strains that diversified from wild populations [7–9]. Recent study has shown that wild mice have diverse and complex immune systems that mimic those of adult humans, whereas laboratory mice have weaker and simpler similar to human neonate-like immune status [10, 11]. In addition, numerous studies reported about the microbiota such as bacteria, fungi, and viruses of wild mice which are necessary for normal physiological and immune function are much different from laboratory strains. In wild mice, their natural micro-organisms results promote host fitness and survival under natural selection pressure and better resemble the immune response of humans. However laboratory mice have poor and more controlled microbiota, and that lack of it and above-mentioned other limitations might decreases the value of laboratory mice to human translational research [12–16].

For these reasons, numerous efforts have been made to develop better mouse strains to overcome the above limitations of laboratory inbred mouse models. As part of these efforts, in Korea, Hallym university research team has been developing a new inbred strain of Korean wild mouse KWM/Hym, subspecies of *Mus musculus* [17]. They have confirmed that KWM/Hym mice are most similar to the PWK/PhJ strain, by their 96% homogeneity 17, 18 and authors concluded that wild derived mice are genetically distinct from common laboratory mice for a number of complex phenotypic characteristics so it would valuable tools for the immunology and biomedical researches. Consequently, in this study, we aimed to determine the visual phenotype of this unique KWM/Hym mouse strain to see their suitability for visual research and experiments.

## Methods

### Mice

The KWM/Hym mice used in this study were provided by the Laboratory Animal Resource Center of Hallym University, Korea. KWM/Hym is an inbred strain that was established from Korean wild mice captured in Chuncheon City, Gangwon Province, South Korea. Mice were kept and inbred at the Laboratory Animal Resource Center of Hallym university, in a conventional animal care facility that maintained a regular environment: 22 ± 2℃, 55 ± 10% relative humidity, and a 12 h light and 12 h night routine cycle. Normal rodent pellet feeds (Cargill Agri Purina, Korea) and water was provided ad libitum. Mice were transferred to Abison Biomedical Research Center (ABMRC) at Yonsei University with animal transferring conditions after each mouse had been tested for their genotype and other analysis. A total of sixteen KWM/Hym mice aged 20–25 weeks, 11 males and 5 females, were used for the vision phenotyping analysis. Also, 22 mice were (4–8 mice per group) used for cataract screening (proceeded at Hallym university, Laboratory Animal Resource Center). All animal experimental procedures are carried out by the guidelines for the Institutional Animal Care and Use of the Laboratory Animals Committee of Yonsei University, College of Medicine, and in accordance with the Yonsei Medical Center Animal Research guidelines, which adhere to the standards articulated in the Association for Assessment and Accreditation of Laboratory Animal Care International (AAALAC) guidelines. All possible steps were taken to avoid animal suffering during the experiments.

### Fundus examination

Mice were anesthetized with an intramuscular injection of zolazepam and tiletamine (30 mg/kg; Zoletil 50^®^, Vibrac, Carros, France) and xylazine (10 mg/kg; Rompun^®^, Bayer animal health) mixture. After anesthesia, pupils were dilated with 0.5% tropicamide and 0.5% phenylephrine mixed eye drops (Mydrin-P, Santen Pharmaceutical Co, Ltd.,). Then, the fundus was examined using Micron^®^ IV (Phoenix Research Labs, Pleasanton, CA, USA), with a wavelength range between 450 and 650 nm, and stored the resultant images in Micron IV StreamPix software (Norpix, Inc., Montreal, QC, Canada).

### Tonometry

Mice were anesthetized as previously described, and intraocular pressure (IOP) was measured using a rebound tonometer (Icare^®^ TONOLAB tonometer, Colonial Medical Supply, Franconia, NH, USA). IOP measurements were performed in the left eye of mice, according to the manufacturer’s instructions. At least 6 IOP readings were obtained, and average data was used for analysis.

### Optical coherence tomography (OCT)

Optical coherence tomography (OCT) imaging was performed using Micron^®^ IV (Phoenix Research Labs, Pleasanton, CA, USA). Mice were deeply anesthetized, pupils were dilated as previously described. After the mice were placed collaterally in front of the OCT camera, we focused the lens on the retina and obtained fundus photographs and retinal OCT scans. Central corneal thickness (CCT) was determined from cross-sectional corneal OCT images that passed through the center of the pupil. We measured the linear distance between the anterior and posterior corneal surfaces in resultant images by the Insight-Animal OCT Segmentation Software (Phoenix Research Labs, USA). Retinal cross-sectional images centered on the optic disc as the main landmark was obtained and we measured the thickness of the retinal layers of KWM/Hym mice. It was measured in two parts, the retinal nerve fiber layer to the outer plexiform layer (RNFL-OPL) and the outer nuclear layer to retinal pigment epithelium (ONL-RPE), distanced 300 μm from the optic nerve head. The thickness of individual retinal layers was determined by the same technique.

### Electroretinography (ERG)

After anesthesia, we performed the full-field electroretinography (ERG) test using Micron Ganzfeld ERG (Phoenix Research Labs, Pleasanton CA, USA) to examine their retinal cells’ function. Before the test, mice were dark-adapted for at least 12 h for scotopic testing and also for sedation. Pupils were dilated with 0.5% tropicamide and 0.5% phenylephrine mixed eye drops (Mydrin-P, Santen Pharmaceutical Co, Ltd.,). Once the pupils were adequately dilated, we applied 2.5% hypromellose (Goniovisc^®^) to lubricate the ocular surface and inserted the electrodes between the eyes, in the middle of the head, and the tail.

First, rod cell function tests (Scotopic ERGs) were recorded in a dark room under dim red illumination, according to the standard protocol provided in the manual. Scotopic ERGs were obtained in response to increasing flash intensities ranging from − 1.7 log cd•s/m2 to 1.9 log cd•s/m2. Then, cone cell function tests (Photopic ERGs) were recorded on the light-adapted mouse in a bright environment with increasing flash intensities ranging from − 0.5 log cd•s/m2 to 4.1 log cd•s/m2.

### Histopathological and structural examination (Hematoxylin and Eosin staining)

After all experiments, mice were sacrificed by asphyxiation with carbon dioxide, and their eyes were dissected immediately. Then, placed the dissected eyes in fixation buffer (65% ethanol, 4% formaldehyde, 5% acetic acid, 3% sucrose) and stored them at 4 °C. The next day, after dehydration, the eyes were embedded in paraffin and sectioned by a thickness of 5–10 nm. Sections were stained with hematoxylin and eosin and checked with a light microscope.

### Transmission electron microscope (TEM)

Eyes were enucleated and fixed for 12 h in 2% Glutaraldehyde and 2% Paraformaldehyde in 0.1 M phosphate buffer (pH 7.4) and washed in 0.1 M phosphate buffer. The anterior segment and vitreous humor were removed. The eyecups with the retina, RPE, and choroid were fixed with 1% osmium tetroxide in 0.1 M phosphate buffer (pH7.4) for 2 h and dehydrated with an ascending ethanol series and embedded with a Poly/Bed 812 kit (Polysciences), polymerized in an electron microscope oven (TD-700, DOSAKA, Japan) at 65℃ for 12 h. 80-nanometer ultrathin sections were cut with an ultra-microtome and placed on copper grids, double stained with 5% Uranyl acetate for 30 min and 3% Lead citrate for 7 min staining, and imaged with a transmission electron microscopy (HT 7800 Tokyo, Japan). All procedures carried out with experts.

### Statistical analysis

All statistical analysis was performed with GraphPad Prism v.5 Software (GraphPad, San Diego, CA, USA). Comparison between groups was performed with the Mann-Whitney *U* test and unpaired t-test. The result of the experiments was presented as mean ± standard error of mean (mean ± SEM). *P* < 0.05 was considered significant. Pearson correlation test and linear regression analysis were used to evaluate the correlation.

## Results

**Fig. 1:**
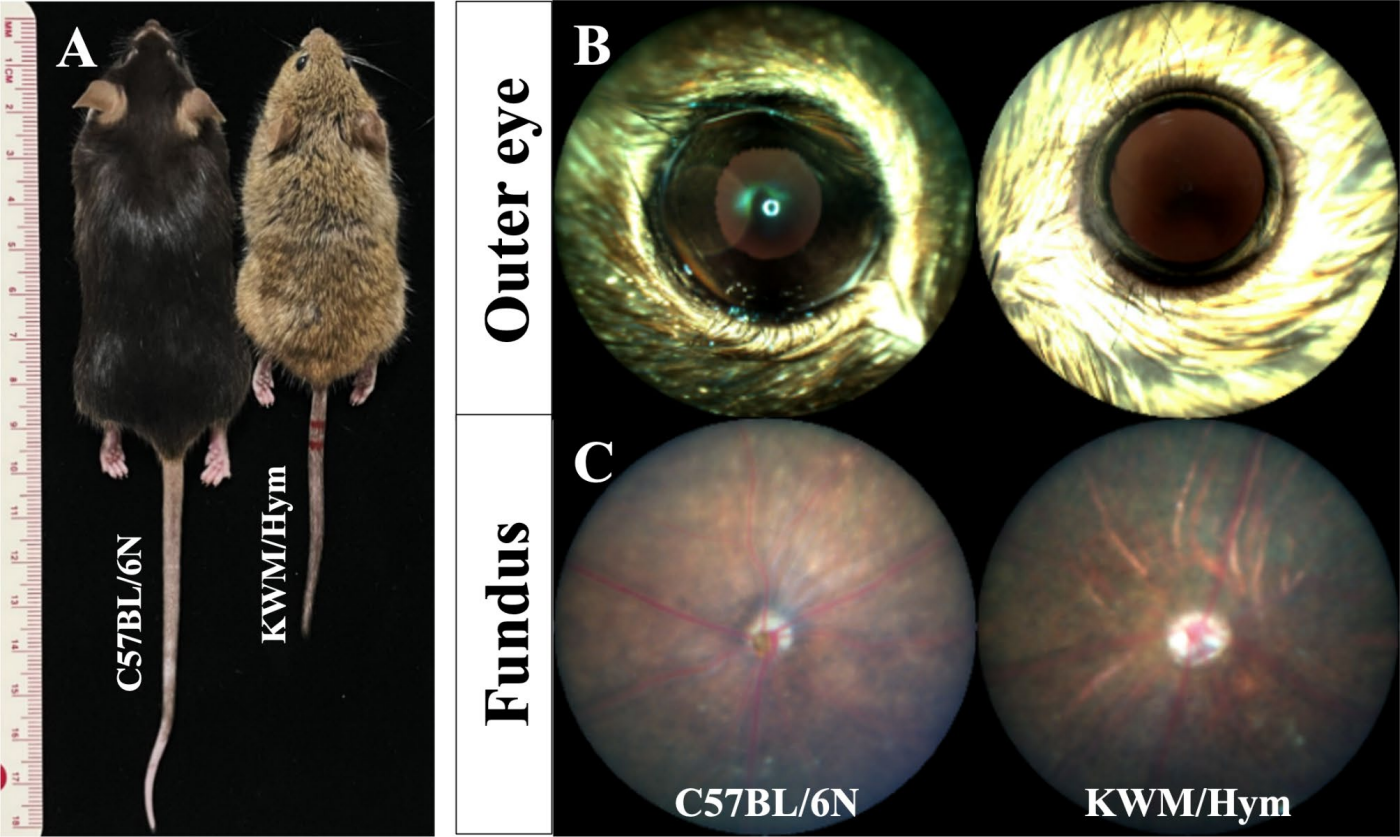
Physical appearance and eye images of C57BL/6 N and KWM/Hym mice. A photograph (**A**) shows comparative appearance of laboratory strain C57BL/6 N (left) and Korean wild mouse KWM/Hym (right). Representative outer eye and fundus images of 25 weeks aged C57BL/6 N mouse (**B**) and KWM/Hym mouse (**C**)

In this study, total thirty-five of Korean wild mice KWM/Hym were analyzed. Sixteen of them were used for visual phenotyping analysis and twenty-two mice were used for additional cataract screening examination. First, as for their appearance, KWM/Hym mice have an agouti-colored fur coat and they were physically smaller than laboratory mice (Fig. 1A). We did not observe any abnormal morphologic changes in the outer eye (Fig. 1B). However, there were crack-like lesions appeared more or less on all KWM/Hym mouse fundus (Fig. 1C). And, when we compared the visual and ocular measurement results of KWM/Hym mice between their age and sex groups, there is no detectable difference between 20 and 25-week- aged groups or male and female groups. Therefore, we summarized their phenotyping data and compared with the age-matched laboratory strain mouse C57BL/6 N which is the most widely used strain in biomedical research.

**Fig. 2:**
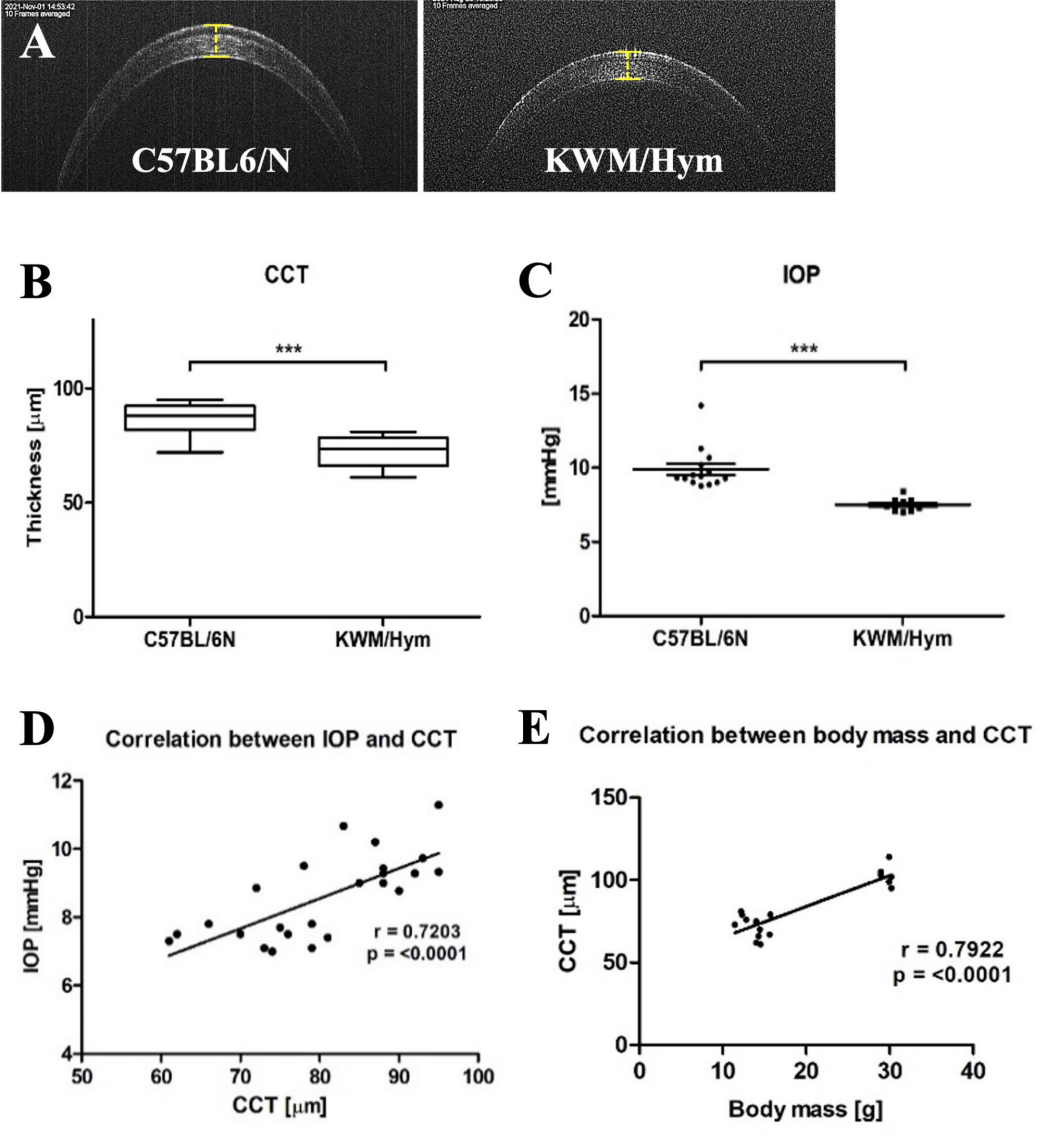
Average CCT and IOP and their correlation. Representative corneal images of C57BL/6 N and KWM/Hym mice (**A**). Comparison of mean CCT value (**B**) and the average IOP (**C**) between two groups. A scatter plot graphs shown a significant positive correlation between CCT and IOP (**D**), and body weight (**E**). CCT, Central corneal thickness; IOP, Intraocular pressure. Control group, *n* = 14; KWM/Hym group, *n* = 13. Values represent mean ± SEM, ***p < 0.001

Visual acuity test was not performed in all mouse due to hyperactivity observed for KWM/Hym. So first, CCT was determined from cross-sectional corneal OCT images that passed through the center of the pupil, and we measured the linear distance between the anterior and posterior corneal surfaces. Cornea was seen as normal but the mean CCT value of KWM/Hym (71.91 μm) was significantly lower than the control group (83.83 μm) (Fig. 2B). Also, an intraocular pressure (IOP) was measured with a rebound tonometer and found significantly lower (*p* = 0.0111) in KWM/Hym (7.50 ± 0.11mmHg) than in C57BL/6 N mice (9.89 ± 0.38 mmHg) (Fig. 2C). Several studies have shown that the corneal thickness is directly correlated with IOP, which means IOP alterations are likely to depend on the corneal thickness or other conditions. In our study, there was a significant moderate positive correlation (*r* = 0.72, *p*-value < 0.0001) between IOP and CCT results (Fig. 2D). To confirm whether the corneal thinness of KWM mice relates to their light body characteristic, we checked their relation. There was also a positive correlation (*r* = 0.79, p- value < 0.0001) between the body mass and CCT (Fig. 2E).

**Fig. 3:**
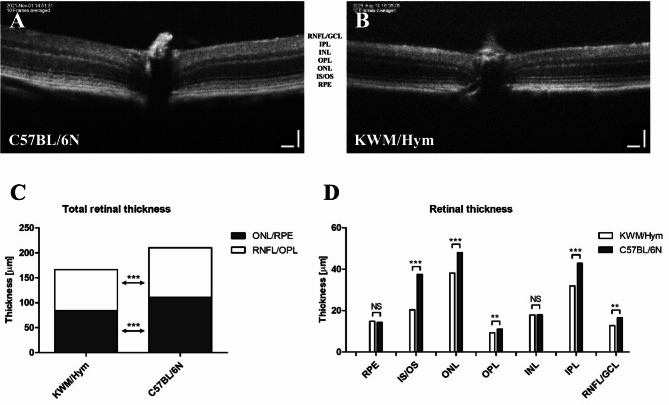
Comparison of the retinal thickness between the C57BL/6 N and KWM/Hym mice. Representative images from the retinal OCT scan of C57BL/6 N (**A**) and KWM/Hym mouse (**B**). Reduced retinal thickness of KWM/Hym mice is shown as totally (**C**) and particularly (**D**). RNFL, retinal nerve fiber layer; IPL, inner plexiform layer; INL, inner nuclear layer; OPL, outer plexiform layer; ONL, outer nuclear layer; IS, inner segment; OS, outer segment; RPE, retinal pigmented epithelium. Scale bar − 100 μm Control group, *n* = 6; KWM/Hym group, *n* = 13. Values represent mean ± SEM, *p < 0,05; **p < 0,01; ***p < 0,001 NS- not significant

Retinal thickness was measured along the cross-sectional OCT image distanced 300 μm from the optic nerve and averaged for two parts from the retinal nerve fiber layer to the outer plexiform layer (RNFL-OPL), and the outer nuclear layer to the retinal pigmented epithelium (ONL-RPE). Following the result obtained with OCT, KWM/Hym mice were characterized by well-ordered retinal layers. However, a significant reduction of retinal thickness was observed in KWM/Hym mice (Fig. 3C) measured as totally (168.0 ± 0.794 μm) and individual sublayers (Fig. 3D), compared to control C57BL/6 N mice (210.6 ± 1.119 μm).

**Fig. 4:**
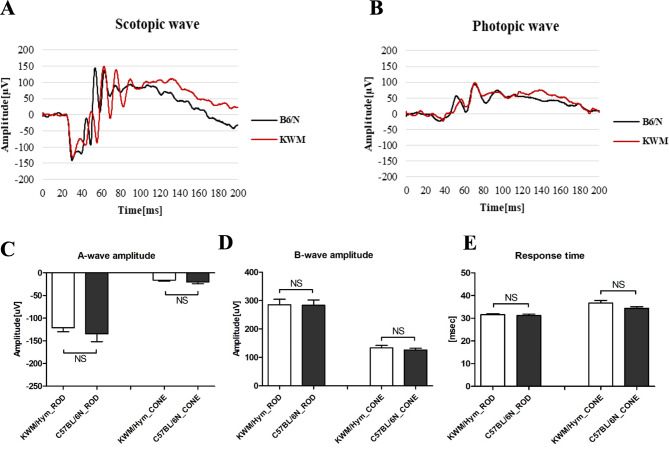
Comparison of the retinal cell function. Representative scotopic (**A**) and photopic (**B**) ERG waveforms obtained from control (black) and KWM/Hym mice (red). Comparison of the average ERG amplitude of A- wave (**C**) indicates the function of the photoreceptors, and B-wave (**D**) indicates the function of bipolar cells in each groups shown no significant differ. An average time of their retinal cells response to the light exposure (**E**) were not different. ERG, Electroretinography. Control group, *n* = 6; KWM/Hym group, *n* = 13. Values represent mean ± SEM, NS- not significant

Next, we assessed the retinal cell function by ERG, recording a- and b-wave amplitudes in both scotopic and photopic conditions with a series of increasing flash intensities. In accordance with results obtained with ERG, there was no difference between A, B-wave amplitudes in dark or light-adapted condition, which means a retinal rod cell (responsible for vision in the dark environment), cone cell (activated in the high light levels), and bipolar cells in KWM/Hym mice showed the same results with control C57BL/6 N mice (Fig. 4C, D) as functionally. Also, in their reaction rate to the light exposure, no significant differences were observed (Fig. 4E).

**Fig. 5:**
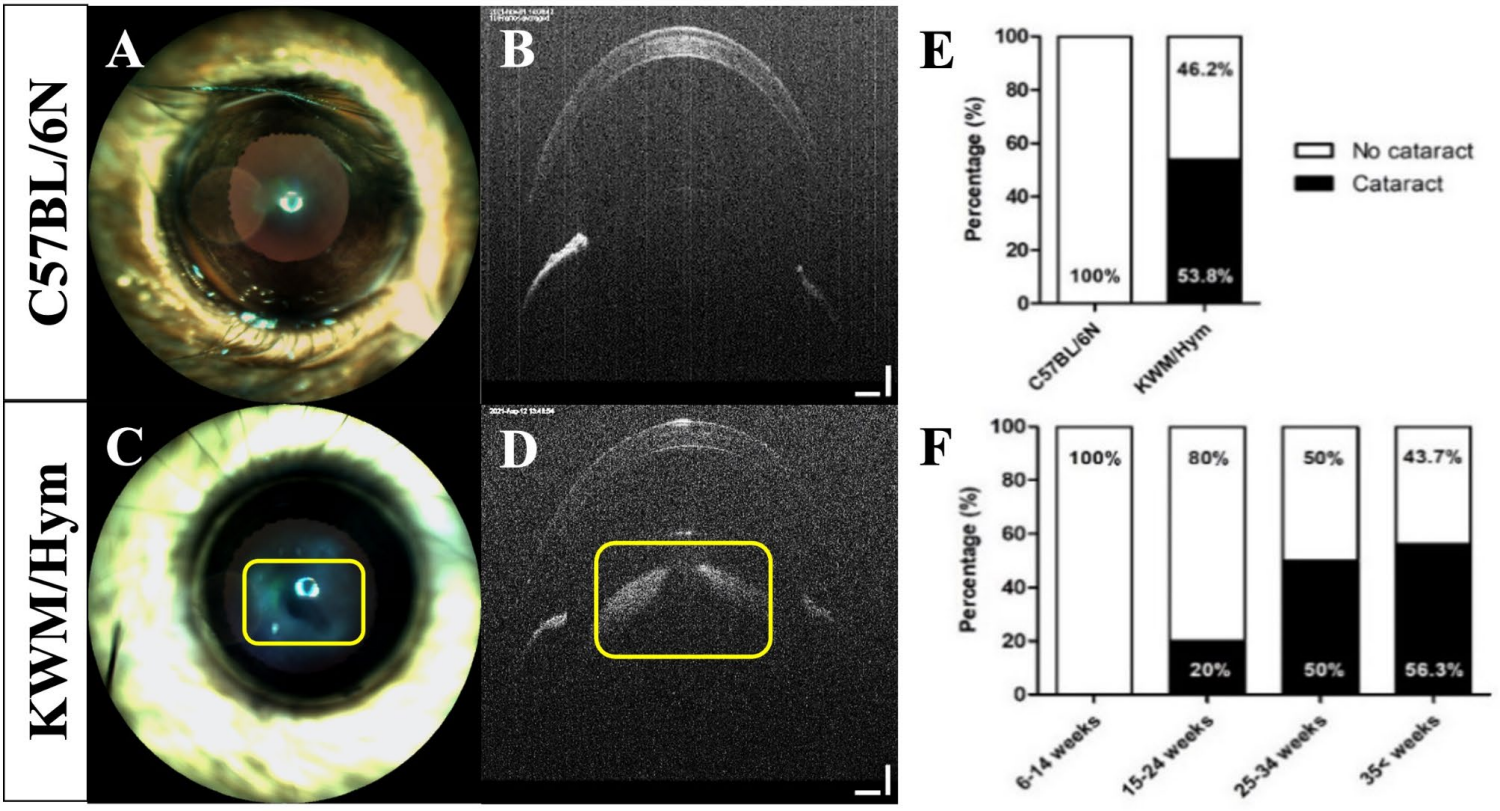
The percentage of lens cataract development in KWM/Hym mice. Representative anterior segment images of 25 weeks aged C57BL/6 N (**A**, **B**) and KWM/Hym mouse (**C**, **D**). The lens opacity in KWM/Hym observed with high reflection (in yellow box). The bar graphs shows the cataract developing percentage in 25 weeks aged laboratory mouse and wild mouse (**E**) and cataract screening result among the different aged KWM/Hym groups (**F**). The cataract developing percentage is calculated by each eye of individual mouse. Control group, *n* = 6; KWM/Hym, *n* = 13 (E); *n* = 4–8 per group (F) Scale bar − 100 μm

Unexpectedly, cataract was observed as frequently among the 25 weeks aged KWM/Hym mice (Fig. 5C, D), whereas the age-matched laboratory strain C57BL/6 N mice developed any abnormal change in the lens (Fig. 5A, B). The lens opacity (yellow box) was detected with a high reflection by the outer eye imaging (Fig. 5C) and anterior segment OCT (Fig. 5D). We found that 53.8% of 25-week-aged KWM/Hym mice had developed the lens cataract (Fig. 5E). Therefore, we examined the lens cataract development percentage in different ages of Korean wild mice. Twenty-two mice were included in this screening and as result, the presence of the cataract started at 15–24 weeks age with 20%. And it reached 50% in only 25–34 weeks, and 56.2% in the over-35-aged group respectively. In addition, cataract was found in 63% of all male mice, and 33% of all female mice in this study.

**Fig. 6:**
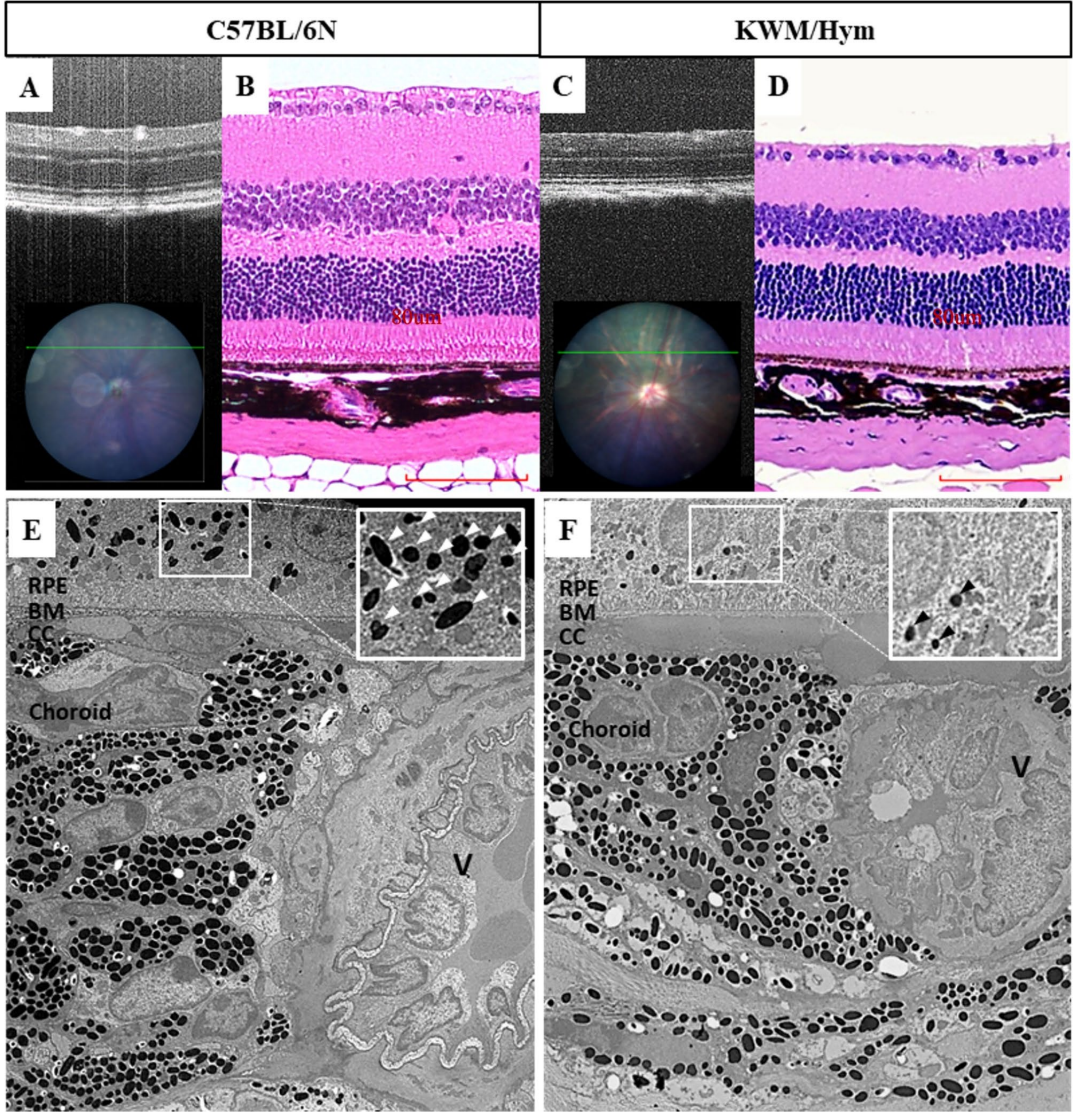
Retinal structure confirmation with histology and transmission electron micrographs. OCT scans show part of the retina section corresponded with the green line on the fundus image (**A**, **C**). Retinal histologic images of 25 weeks-aged laboratory strain C57BL/6 N (**B**) and KWM/Hym mouse (**D**) show their retinal well-organization. Transmission electron micrograph shown an ultrastructure of retina-choroidal section (**E**, **F**). Melanosomes (arrowheads) appeared with great reduction in RPE layer of KWM/Hym (**F**) compared to control strain (**E**). RPE, Retinal pigmented epithelium; BM, Bruch’s membrane; CC, choriocapillaries; V, large vessel in choroid. Scale bar – 80 μm, TEM- 15x magnification

**Fig. 7:**
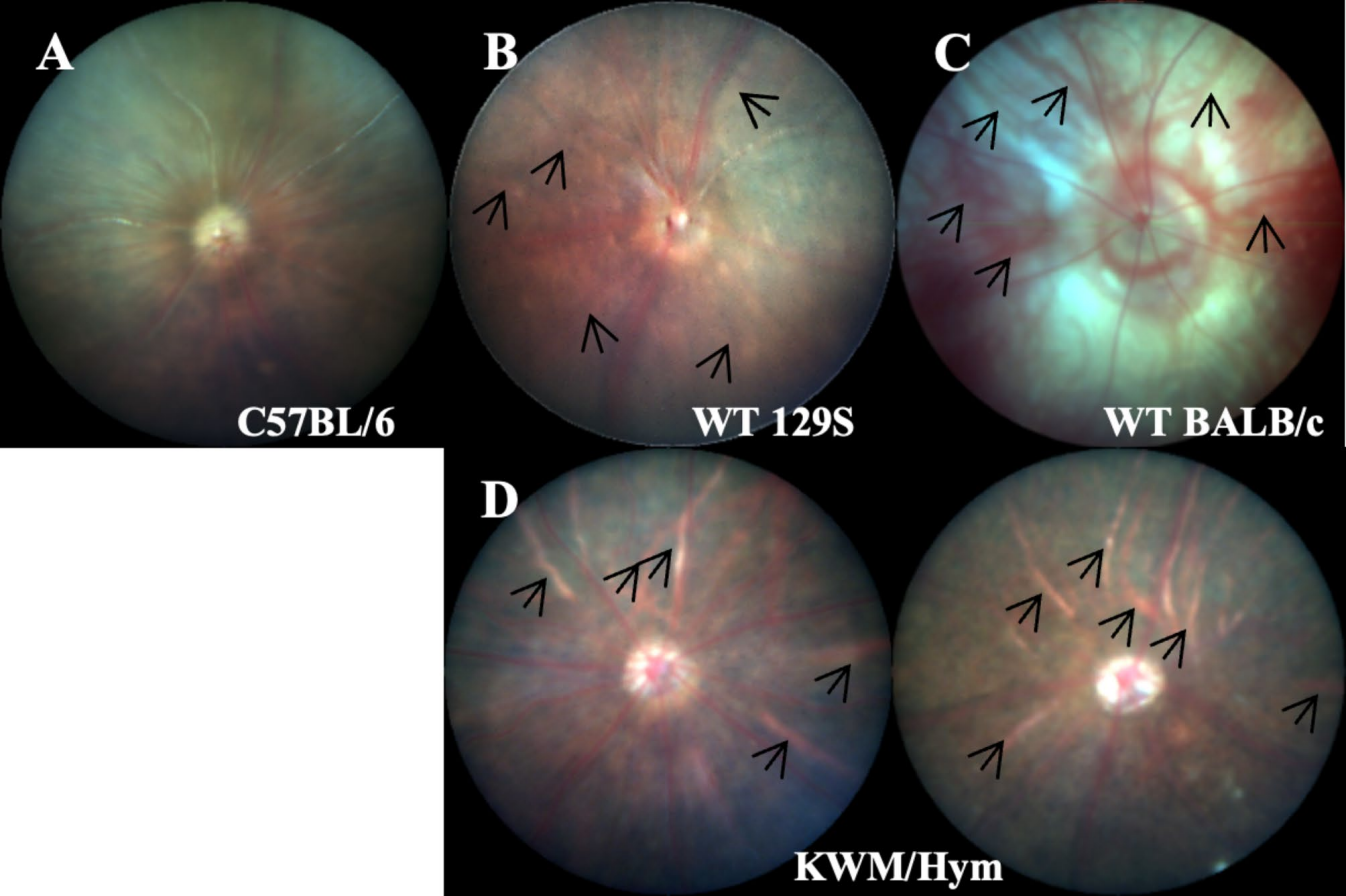
The fundus images of the wild-type mice strains. Choroidal posterior medium and large vessels (black arrows) shown in hypopigmented mice (**B**-**D**) compared to C57BL/6 N mouse fundus (**A**). WT, Wild-type mouse

As for the changes in fundus, we could not find any abnormality from OCT scans corresponded with those changes (Fig. 6C). Therefore, we proceeded with a retinal histopathological and TEM test to reveal their retinal structure more clearly. Mice were sacrificed by asphyxiation with carbon dioxide, and their eyes were dissected immediately. Tissue preparation and experimental procedures were carried out by experts. In consequence, the retinal histology image of the KWM/Hym mice showed well-ordered retinal layers (Fig. 6D) and characterized by their slightly hypopigmented RPE. Also, through an electron micrograph, this characterization is presented and confirmed in their ultrastructure (Fig. 6E, F) with reduced light-absorbing melanosomes (black arrowheads) compared to the control C57BL/6 N mouse which shown numerous melanosomes (white arrowheads). Also, by an electron microscopic examination, there were no abnormalities or damages observed in their Bruch’s membrane (BM) and choroid because those changes of the following structures can show a similar fundus appearance (crack-like changes) as KWM/Hym mice. Since there were no abnormal changes confirmed in the retinal structure of KWM/Hym mice by the above experiments, we compared their fundus image with other wild-type laboratory strains (Fig. 7). So, we considered that KWM/Hym mice showed a lighter fundus and more apparent choroidal vasculature (Fig. 7D) as similar to other bright-colored strains such as 129 S (Fig. 7B) which has a light-brown fur coat, and albino BALB/c mice (Fig. 7C) compared to black-coated C57BL6 mouse fundus (Fig. 7A).

## Discussion

Although the usage of a wild-derived mouse in biomedical research is under-researched, the current studies showed the great potential of wild mice as mentioned in the introduction, and they suggested that wild mice would add a valuable component to existing experimental mouse resources. As part of the efforts to develop a better mouse model, our team introduced a new inbred strain of KWM/Hym derived from a wild population in Korea. In the present study, we aimed to analyze the visual phenotype of these unique mice to see whether they are suitable for visual experiments.

Wild and wild-derived mice such as PWK/PhJ, CAST/EiJ, and WSB/EiJ body weight and some of their comparable eye length and retinal characteristics previously mentioned in other studies [19, 20]. KWM/Hym mice used in this study were also smaller as reported [17]. So the thinness of the cornea and retina of KWM/Hym mice can be explained by their light body weight characteristics. In previous studies, they have shown a positive correlation between body weight and measured corneal thickness [21] and eye mass [22]. Also, we considered that according to their thin cornea, intraocular pressure was measured dramatically lower than in laboratory strain. Numerous studies have reported that corneal thickness is directly correlated with intraocular pressure, which means pressure alterations are likely to depend on the corneal thickness or other conditions [23–25]. Similarly, in our study, a significant positive correlation confirmed this relation. Moreover, wild mice have good vision. Their well-developed a- and b-waves in electroretinogram indicated their normal visual function and confirmed they would be useful in the visual experiment.

In the mammal retina, the choroid and the retinal pigmented epithelium cell layers contain numerous melanosomes and pigment granules that absorb excess light entering the eye. Pigment density differs in various parts of the retina, which can give the fundus a mottled appearance when viewed with the ophthalmoscope [26]. Typically, the wild-type C57BL/6 N controls have a uniformly black fur coat and carry numerous darkly pigmented melanocytes in their retinal pigmented epithelium and choroid. Through this study, the retinal ultra-structural analysis confirmed these agouti-colored KWM/Hym mice had comparatively fewer melanocytes in their retina. Therefore, we considered that due to their hypopigmented retinal phenotype, they showed a lighter fundus and more apparent choroidal vasculature as similar to other bright-colored strains such as 129 S which has a light-brown fur coat and albino BALB/c mice compared to wild-type C57BL6 mouse fundus.

Cataracts were observed with high frequency among the 25 weeks aged KWM/Hym mice and confirmed by additional cataract screening in different age groups of Korean wild mice, whereas wild-type laboratory strains develop the cataracts naturally when aged out to 12–30 months [27, 28]. At present, there are several mouse models of cataracts, but most of them are genetic mouse cataract models provided rather to the study of lens development or early onset lens defects than the aging process [29]. Furthermore, other cataract models, are induced with ultraviolet radiation [30] or chemical such as sodium selenite that induces acute oxidative stress, triggering cataract formation within only 4–6 days after injection [31, 32]. Although these models are suitable for studying severe cataracts, they do not capture the gradual and variable onset that occurs with aging. On the other hand, even various strains of wild-type mice develop age-related cataracts naturally up to 30 months of age and show various opacities [27], but it is over-priced and difficult to keep mice out to such old age. That reflects the KWM/Hym mouse might be beneficial in human cataract studies. To enhance the applicability of the KWM/Hym model to human cataracts, it is necessary to investigate variations in cataract-related biomarkers (such as reactive oxygen species, superoxide dismutase, catalase) and mutations in genes (such as Crystallin Alpha A, Crystallin Alpha B) associated with cataract formation, as reported in humans, using the KWM/Hym model.

In contrast to the higher prevalence of cataracts in women in humans, the prevalence of cataracts was higher in males in KWM/Hym mice. According to cataract surgery statistics, the incidence of cataracts requiring surgery is higher in women in their fifties to seventies but is higher in men in earlier or later age groups [33]. The higher frequency of cataract surgery in postmenopausal women is because the protective effect of 17 beta-estradiol against oxidative stress caused by hydrogen peroxide is reduced [34]. The typical sexual maturation of laboratory mice is 3 to 6 months of age, and the age of menopause in humans is 9 months of age [35]. In KWM/Hym mice, the onset of cataracts at 25 weeks of age corresponds to before the perimenopausal period. Therefore, the higher prevalence in male KWM/Hym mice is thought to be due to the protective effect of estrogen against oxidative stress, and we plan to confirm this in future studies.

## Conclusions

By providing visual experiments in our study, we suggest that KWM/Hym mouse which is available as a request to the Department of Medical Genetics at Hallym University College of Medicine is useful for vision research. Retina, intraocular pressure and external eye morphology present stable ranges. Therefore, we suggest that KWM/Hym mouse could be an animal model for human eye disease.

## Data Availability

Data of the study are available from the corresponding author on reasonable request.

## Abbreviations

OCT: Optical coherence tomography
CCT: Central corneal thickness
RNFL-OPL: Retinal nerve fiber layer to the outer plexiform layer
ONL-RPE: Outer nuclear layer to retinal pigment epithelium
ERG: Electroretinography
TEM: Transmission electron microscopic
IOP: Intraocular pressure
BM: Bruch’s membrane
